# Development and characterization of a microfluidic model of the tumour microenvironment

**DOI:** 10.1038/srep36086

**Published:** 2016-10-31

**Authors:** Jose M. Ayuso, María Virumbrales-Muñoz, Alodia Lacueva, Pilar M. Lanuza, Elisa Checa-Chavarria, Pablo Botella, Eduardo Fernández, Manuel Doblare, Simon J. Allison, Roger M. Phillips, Julián Pardo, Luis J. Fernandez, Ignacio Ochoa

**Affiliations:** 1 Group of Structural Mechanics and Materials Modelling (GEMM), Centro Investigacion Biomedica en Red. Bioingenieria, biomateriales y nanomedicina (CIBER-BBN), Spain; 2 Aragón Institute of Engineering Research (I3A), University of Zaragoza, Spain; 3 Aragon Institute of Biomedical Research, Instituto de Salud Carlos III, Spain; 4 Aragón Health Research Institute (IIS Aragón), Biomedical Research Centre of Aragón (CIBA), Zaragoza, Spain; 5 Dpt. Biochemistry and Molecular and Cell Biology, University of Zaragoza, Zaragoza, Spain; 6 Centro Investigacion Biomedica en Red. Bioingenieria, biomateriales y nanomedicina (CIBER-BBN), Spain; 7 Bioengineering Institute, University Miguel Hernández, Spain; 8 Instituto de Tecnología Química (Universitat Politècnica de Valencia-Consejo Superior de Investigaciones Científicas), Spain; 9 Department of Biology, University of Huddersfield, Queensgate, Huddersfield HD1 3DH, United Kingdom; 10 Department of Pharmacy, University of Huddersfield, Queensgate, Huddersfield HD1 3DH, United Kingdom; 11 Dpt. Microbiology, Preventive Medicine and Public Health, University of Zaragoza, Zaragoza, Spain; 12 Aragón I+D Foundation (ARAID), Government of Aragon, Zaragoza, Spain

## Abstract

The physical microenvironment of tumours is characterized by heterotypic cell interactions and physiological gradients of nutrients, waste products and oxygen. This tumour microenvironment has a major impact on the biology of cancer cells and their response to chemotherapeutic agents. Despite this, most *in vitro* cancer research still relies primarily on cells grown in 2D and in isolation in nutrient- and oxygen-rich conditions. Here, a microfluidic device is presented that is easy to use and enables modelling and study of the tumour microenvironment in real-time. The versatility of this microfluidic platform allows for different aspects of the microenvironment to be monitored and dissected. This is exemplified here by real-time profiling of oxygen and glucose concentrations inside the device as well as effects on cell proliferation and growth, ROS generation and apoptosis. Heterotypic cell interactions were also studied. The device provides a live ‘window’ into the microenvironment and could be used to study cancer cells for which it is difficult to generate tumour spheroids. Another major application of the device is the study of effects of the microenvironment on cellular drug responses. Some data is presented for this indicating the device’s potential to enable more physiological *in vitro* drug screening.

A characteristic feature of solid tumours is their unique physiological and biological microenvironment, which consists of multiple cell types and gradients of oxygen tension, nutrients and waste products which vary as a function of distance from a supporting blood vessel^1^,^2^,^3^,^4^,^5^. This tumour microenvironment has significant biological and therapeutic implications including the promotion of a more aggressive cancer phenotype and increased cellular resistance to radiotherapy and chemotherapy^6^,^7^,^8^. In the search for novel therapeutics, the use of more physiologically relevant experimental models that can mimic key aspects of the tumour microenvironment *in vitro* is required^9^,^10^,^11^. One of the models that is currently used is the three dimensional multicellular spheroid, however, this model also has a number of key limitations: (i) some cell lines do not form spheroids; (ii) although spheroid size can be controlled, cell density within a spheroid cannot; (iii) controlling the extracellular matrix (ECM) within a spheroid is not possible; and (iv) direct visualization of cells within the microenvironment created by the spheroid is difficult in real-time due to the thickness of the viable rim of the spheroid (typically a few hundred microns)^10^,^12^. Analysis of the effects of the spheroid microenvironment on tumour cell biology and drug response typically requires fixation and sectioning of spheroids^13^ or cell disaggregation by sequential disaggregation of the spheroid^14^. Although laser confocal microscopy can be used to visualize spheroids in real-time, this technique has a maximum depth penetration of approximately 50 μm, which is not enough to visualize cells within the hypoxic region of spheroids^15^. Other techniques such as light sheet microscopy could increase this visualization depth but these techniques are technically challenging and not widely available^16^. There is therefore a need to develop and validate new experimental models of the tumour microenvironment.

In this context, microfluidic systems have emerged as a potential way of recreating important aspects of the tumour microenvironment *in vitro* and analysing cellular effects in real-time. These systems have been used to visualize cellular processes in real-time such as tumour cell chemotaxis, angiogenesis, tumour cell extravasation, tumour-stroma cross-talk and cellular responses to drugs^17^,^18^,^19^,^20^,^21^,^22^,^23^. However, the focus of most microfluidic research remains within the engineering field, requiring highly specialist equipment and resources for microdevice fabrication (for example clean room processing, slow manufacturing processes and in-depth knowledge of fluid dynamics)^24^. Microdevices that are easier to fabricate and operate will encourage the more widespread adoption of microfluidic devices in biomedical and pharmacological research. This article presents an easy-to-operate microdevice which can mimic the three dimensional architecture of multicellular spheroids, whilst at the same time generating a visible, live “tumour slice” that allows easy monitoring of cells in different regions of the microenvironment in real-time as well as their response to different drugs. This model also has the potential to assess the ability of drugs to penetrate through several cell layers which can be a major barrier to effective drug treatment^25^.

The microdevice comprises a central microchamber flanked by two lateral microchannels separated by a series of projections. This design has been shown to be robust and versatile, since it allows for liquid confinement in the central microchamber without invading the lateral microchannels^19^,^26^,^27^. For this study, tumour cells were embedded within a collagen hydrogel thereby mimicking the ECM, and confining cells to the central microchamber. The lateral microchannels were used to perfuse different media or compounds and due to the configuration of the central chamber, normoxic, hypoxic and necrotic regions were naturally generated. Colon and Glioblastoma tumour cell behaviour in different regions of the microdevice were studied and analysed in conjugation with measurements of hypoxia and glucose concentrations across the device. The potential of this technology for analysing the impact of microenvironmental parameters on drug response is exemplified by the differential cellular response to several well-known drugs in different parts of the microdevice.

## Results

### Microdevice operation and visualization of cells

**Figure 1:**
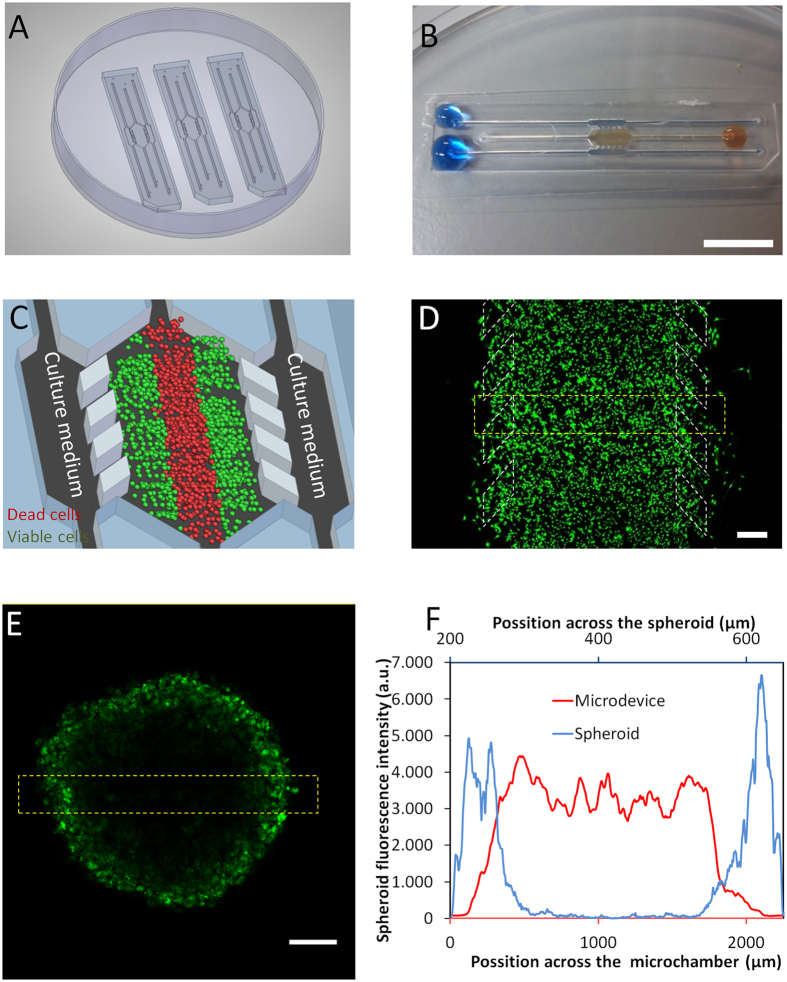
Microdevice operation and modelling of the tumour microenvironment. (**A**) Appearance of the microdevice. Microdevices are fabricated by injection moulding and can be attached to the bottom of a Petri dish using biocompatible adhesive; three identical devices are shown. (**B**) Magnified image of a microdevice with collagen hydrogel confined to the central microchamber and blue-coloured water perfused through the two lateral microchannels to ease visualization. Droplets are located on top of the inlets to prevent evaporation. Scale bar is 1 cm. (**C**) The principle of the live ‘tumour slice’: Culture medium perfused through the lateral microchannels provides nutrients and oxygen creating physiological gradients across the device. Cells near the ‘surrogate’ blood vessels are viable, whereas oxygen-poor cells in the centre of device start to die creating a ‘necrotic core’ similar to the necrotic regions of tumours. (**D**) Cellular visualization in the microdevice. 20 million U-251 MG cells/ml were embedded in collagen and pipetted into the central chamber of the microdevice. Image shows appearance by confocal microscopy 24 h later. Cells were labelled before injection with the green-fluorescent lipid dye Dio Vybrant® which stains cell membranes enabling all cells to be visualized. Scale bar is 400 μm. (**E**) Incomplete cell visualization within a multicellular spheroid due to its thickness. 10000 U-251 MG cells were labelled with green-fluorescent Dio Vybrant® dye, in suspension to ensure all cells were equally labelled and these were used to form the spheroid. Scale bar is 400 μm. (**F**) Quantification of cellular fluorescence across the yellow bordered regions in the microdevice and the spheroid as indicated in (**D,E**).

Polystyrene-based microdevices were fabricated by injection moulding, and attached to Petri dishes using biocompatible adhesive (Fig. 1A). The microdevice was designed so that each of the microchambers could be individually loaded with liquid without liquid spreading to adjoining chambers, allowing for the development of a ‘3D cell culture’ system with injected hydrogel confined to the central chamber (Fig. 1B). The lateral microchannels remained hydrogel-free and effectively served as ‘surrogate blood vessels’ for the delivery of nutrients, oxygen, NK cells and drugs as required (Fig. 1C). For both the microdevice and the spheroid experiments shown in Fig. 1D,E, adherent U-251 MG cells were trypsinized and after detachment were labelled with Vybrant® Dio in suspension in order to fluorescently label the entire cell population. Following 3 days of incubation in the microdevice, Vybrant® Dio labelled U-251 MG cells were clearly visible throughout the central chamber of the microdevice (Fig. 1D). In contrast, for multicellular spheroids formed from Vybrant® Dio labelled U-251 MG cells, only the cells on the periphery of the spheroid were visible by confocal microscopy (Fig. 1E,F and Supporting Fig. 2). This indicates one of the key limitations of the multicellular spheroid *versus* the microdevice, with Vybrant® Dio labelled U-251 MG cells only being visible on the spheroid periphery despite there being fluorescent cellular labeling throughout.

### Analysis of fluid flow

**Figure 2:**
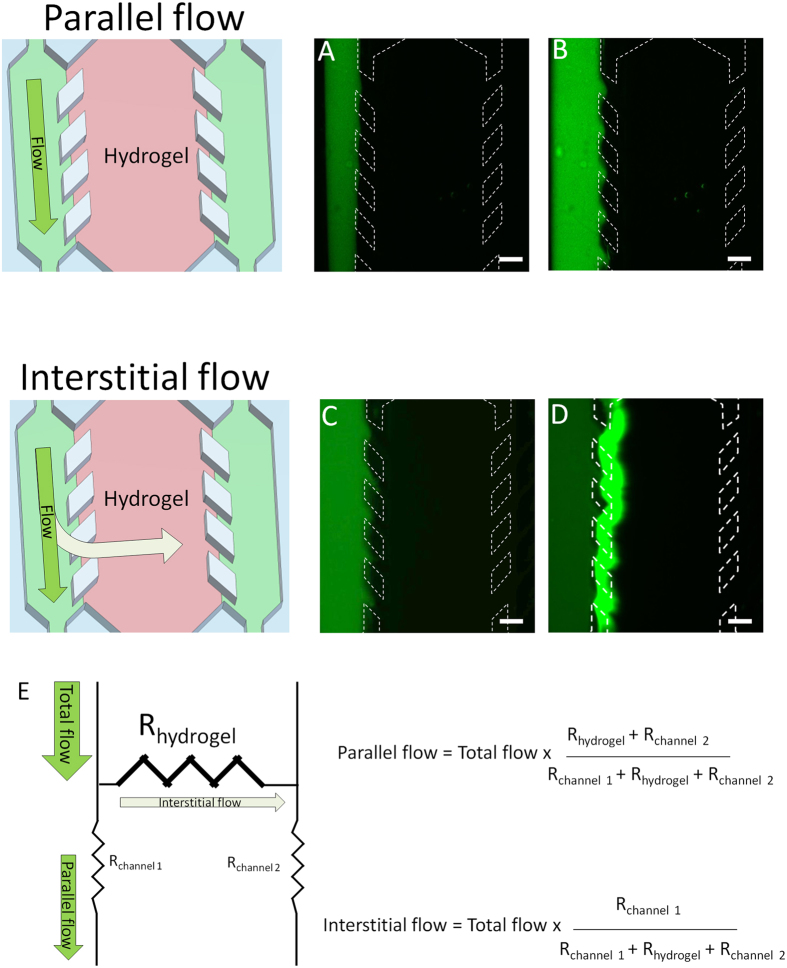
Flow characterization. After collagen hydrogel polymerization, green fluorescent beads were suspended in PBS and perfused through the left lateral microchannel. (**A,B**) When low volumes (10 μl) were injected, the flow profile observed was completely parallel to the hydrogel and no sphere penetration into the hydrogel was observed. (**C,D**) When large volumes (300 μl) were injected, an interstitial flow through the hydrogel was observed, showing sphere penetration into the hydrogel. Scale bar is 400 μm. (**E**) Parallel and interstitial flow are determined by the indicated parameters, where “R_hydrogel_” is the flow resistance generated by the hydrogel, and “R_channel_” is the flow resistance through the lateral microchannels.

After loading the central chamber of the device with hydrogel (without cells) and allowing time for its complete polymerization, 10 μl of cell culture media containing 0.2 μm green-fluorescent polystyrene beads was injected by manual pipetting through one of the lateral microchannel. Using fluorescence time-lapse microscopy, the flow profile was analysed and observed to be completely parallel to the central microchamber with no interstitial flow into or through the hydrogel located in the central microchamber (Fig. 2AB and Supporting movie 1). This indicates the high flow resistance generated by the collagen hydrogel confined in the central microchamber. However, when volumes of 300 μl were injected through one of the lateral microchannels, a clear interstitial flow was observed into the central microchamber although the fluorescent sphere front did not reach the opposite lateral microchannel (Fig. 2C,D and Supporting movie 2). The hydrogel exerted a high flow resistance, causing that major part of the fluid to flow through the lateral microchannel (Fig. 2E). Given that the central microchamber has an internal volume of 2 μl, this suggests that less than 1% of the injected volume (300 μl) was entering the hydrogel. This suggests that in order to completely replace or replenish the medium inside the hydrogel a large volume of medium (e.g. 1–2 ml) should be injected. These results show that by simply altering the volume of solution manually pipetted through one of the lateral microchannels, it is possible to generate parallel flow or interstitial flow as desired without the need for any additional equipment or pumps.

### Cell viability analyses

U-251 MG cells were embedded in collagen in the central microchamber of the device at different cell densities and were cultured for several days. This was to determine whether a necrotic region was formed within the central microchamber, which is typical of the tumour microenvironment *in vivo*. After three days, for cells embedded at the highest cell density (40 million U-251 MG cells/ml) a distinct central region or ‘belt’ of cells lacking viable calcein (CAM) staining and positive for propidium iodide (PI) was observed (Supporting Fig. 3). Positive PI staining indicates loss of cell membrane integrity and is routinely used to detect cellular necrosis and cells in the late stages of apoptosis as well as several other forms of cell death. In contrast, microdevices initially seeded with 4 or 10 million U-251 MG cells/ml showed no significant PI staining by 3 days.

**Figure 3:**
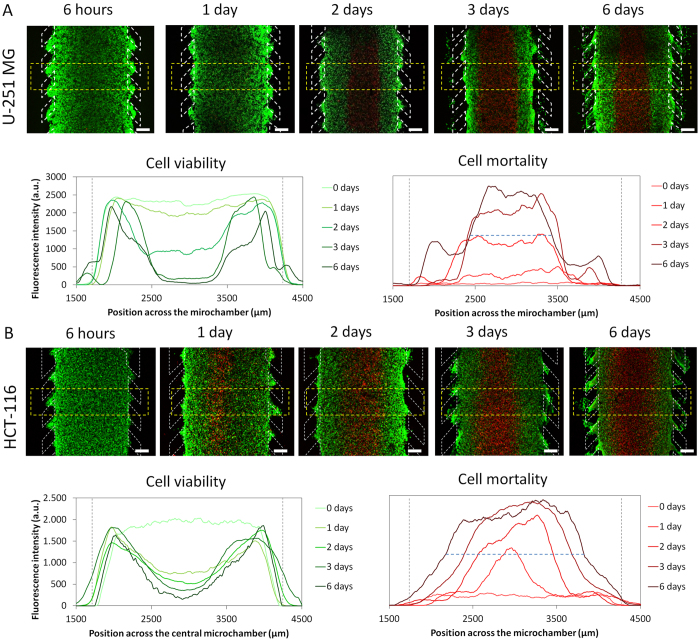
Necrotic core generation within the microdevice. U-251 MG and HCT-116 cells were embedded in collagen hydrogel in the central microchamber. (**A**) 40 million U-251 MG cells/ml were confined in the central microchamber and cell viability was evaluated at the indicated times using calcein (CAM) to stain viable cells green and propidium iodide (PI) to stain dead cells red. The graphs show CAM or PI fluorescence intensity profile along the delimited region in the images. Position of the pillars is delimited by a grey dashed line. (**B**) HCT-116 cells evaluated under identical experimental conditions showed faster necrotic core generation. The width of the necrotic core after 6 days was measured as the distance between those positions in the microchamber that reached 50% of the maximum PI fluorescence intensity (blue dashed horizontal line). Necrotic core width for U251-MG cells was 1011 ± 12 μM, and for HCT-116 cells was 1643 ± 9 μM, p-value < 0.05. Scale bar is 400 μm.

To study the kinetics of ‘necrotic core’ formation, U-251 MG and HCT-116 cell viability was evaluated at different time points after introducing cells into the central chamber. In experiments using U-251-MG cells, cells staining positive for PI gradually appeared in the centre of the microchamber over the first 3 days. Inversely, the thickness of the viable region, as indicated by CAM staining, decreased as time increased (Fig. 3A). The system stabilized after 3 days, and no further changes were observed in either the viable or necrotic region after longer culture times of up to 6 days (Fig. 3A, graphs). The experiment was repeated using HCT-116 cells, with fluorescence time-lapse microscopy showing a clear, central ‘necrotic region’ appearing by 24 hours which was significantly faster than for the U-251 MG cells (Fig. 3B and Supporting Movie 3). Additionally, the HCT-116 necrotic core generated was significantly larger in size than that of the U-251 MG cells at 6 days (1643 ± 9 microns versus 1011 ± 12 microns respectively).

### Cell proliferation analyses

**Figure 4:**
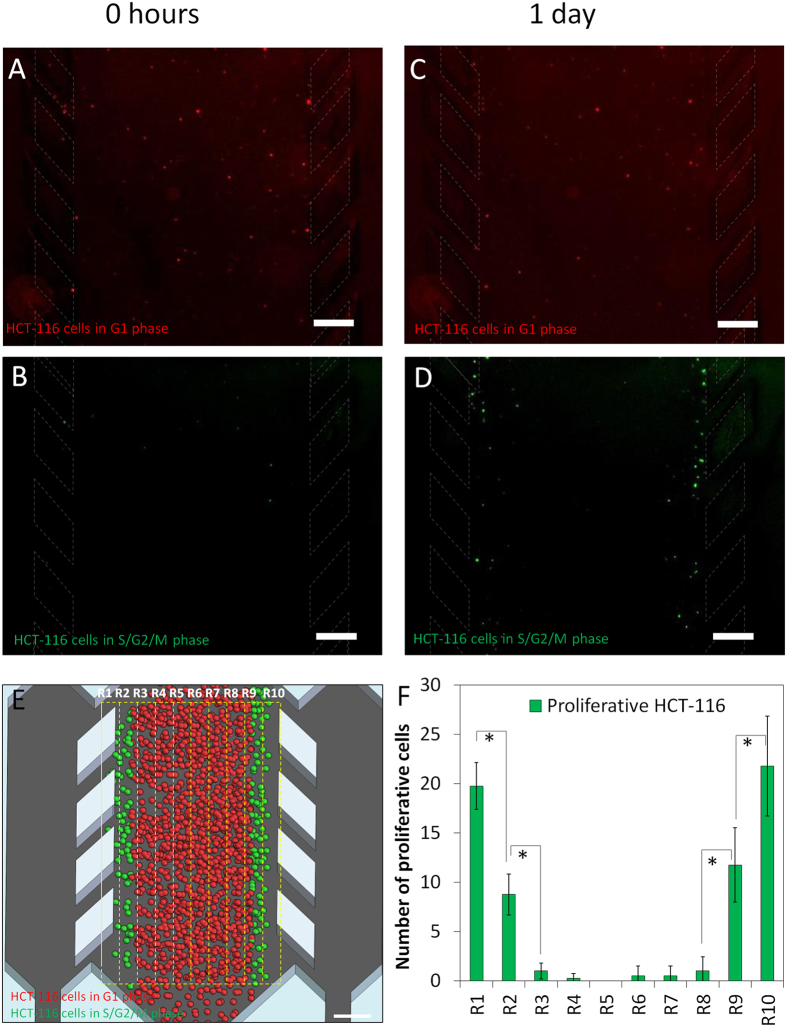
HCT-116 Cell proliferation. 40 million HCT116 cells/ml were confined to the central microchamber; 5% of these cells were transduced with the cell cycle sensor Premo FUCCI®, which stains G_0_/G1 phase cells in red and S, G2 and M phase cells in green. (**A,B**) After collagen polymerization, red staining cells (G1 phase or quiescent) were randomly distributed through the central microchamber, whereas only few cells stained green (S, G2 or M phase cells). (**C,D**) After 24 hours, an increase in green staining cells was clearly observed near the lateral microchannels indicating cell proliferation. (**E,F**) The central microchamber was vertically divided into ten regions (200 μm steps) and proliferation was quantified as the number of green cells observed in each region at 24 h compared to 0 h. Scale bar is 400 μm.

The formation over time of a central ‘necrotic core’ furthest from the peripheral channels suggested the development of gradients of nutrients and oxygen across the chamber. The assumption was that cells close to the peripheral channels should be well nourished and oxygenated, whereas cells some distance away from these ‘surrogate blood vessels’ would suffer from nutrient and oxygen deprivation. To study possible effects this might have on cell proliferation in addition to the observed effects on cell survival, both U-251 MG and HCT-116 cell lines were transiently transduced with FUCCI cell cycle fluorescent reporters. These fluorescent reporters change colour depending on the position of the cell in the cell cycle with quiescent and G1 cells fluorescing red, cells progressing from G1 into S phase fluorescing yellow and proliferative cells in S, G2 or M phase fluorescing green. In order to more easily visualize active cell cycling and proliferation in real-time, only 5% of the total cells were transduced, whereas the other 95% of the cells remained unstained. A few minutes after injecting HCT-116 cells into the microdevices, only a few ‘green’ cells were observed, whereas a higher number of ‘red’ cells were detectable, both randomly distributed across the chamber. This was consistent with a higher proportion of the cell population being in G1 phase (Fig. 4AB). Time-lapse fluorescence microscopy showed that after 1 day in the microdevice, only those cells located near to the lateral microchannels were actively proliferating and ‘green’ S phase or G2/M phase cells could only be detected here (Fig. 4C–F). These results combined with the cell viability analysis described earlier demonstrate that a proliferative outer layer of cells is generated followed by a slower-proliferating or quiescent middle region and eventually a necrotic core as distance from the ‘surrogate blood vessel’ increases. In this way, the microdevice appears to create a ‘visible’ microenvironment that is similar in aspects to the actual tumour microenvironment. In contrast to multicellular spheroids, the microdevice generates a visual ‘tumour slice’ through the tumour microenvironment enabling direct visualisation of cells within the microenvironment. Interestingly, when this experiment was repeated using U-251 MG cells, no S or G2/M phase cells staining green were observed after 24 hours, whereas G1 phase cells staining red were randomly distributed (Supporting Fig. 4).

### Oxygen and glucose gradients within the microdevices

**Figure 5:**
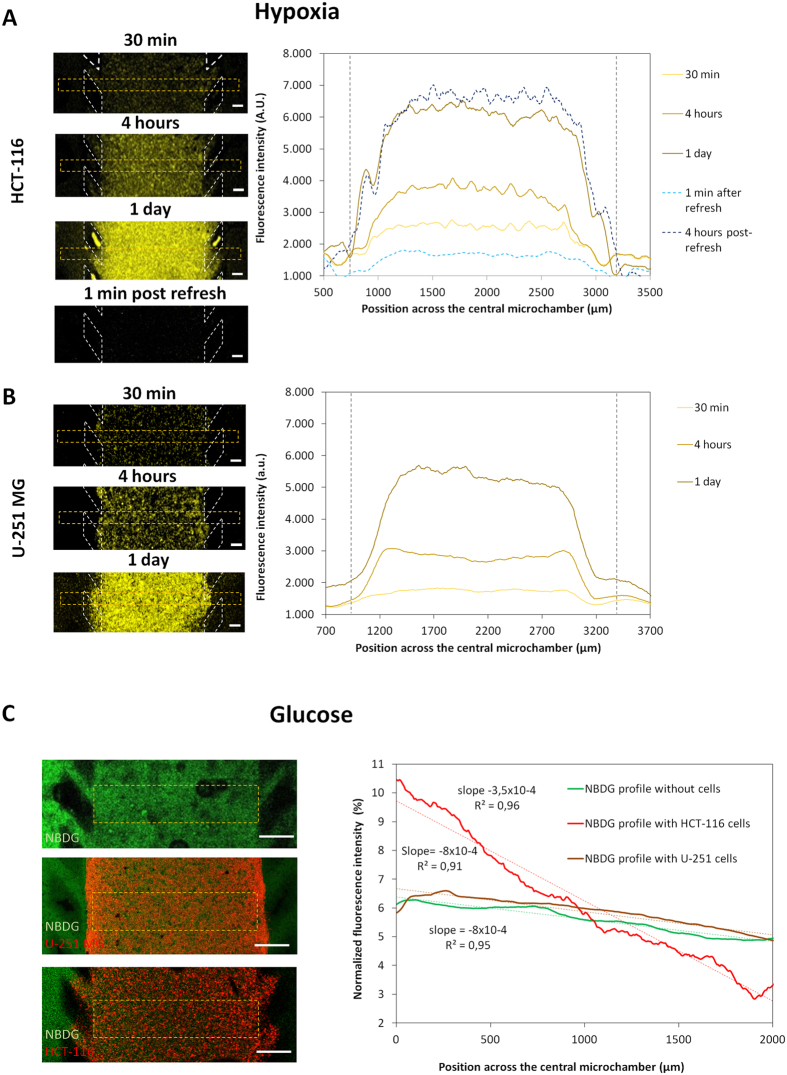
Oxygen and glucose profile. HCT-116 and U-251 MG cells were embedded within collagen hydrogel and cultured in the central microchamber. (**A**) A hypoxia-sensing dye was added to the hydrogel/cell mixture prior to injection within the microdevice to monitor and quantify the formation of the hypoxia gradient through the experiment in real-time. After collagen polymerization, culture medium containing the dye was added to the lateral channels to keep the dye concentration constant through the experiment. The left panels show hypoxia-induced fluorescence intensity in the central microchamber increased during the experiment as well as how the hypoxia signal disappeared when medium inside the hydrogel was ‘refreshed’ by interstitial flow. The graph shows the hypoxia-induced fluorescence profile across the central microchamber. The levels of hypoxia rapidly increased across the first 300 μm and reached a plateau in the centre of the microchamber. 1 ml of fresh media perfused through one of the lateral microchannels, reduced hypoxia-induced fluorescence to initial ‘normoxic’ values. 4 hours post-refresh, the hypoxia-induced fluorescence reached again the values observed before. Scale bar is 200 μm. Position of the pillars is delimited by a grey dashed line. (**B**) The same experiment was repeated for U-251 MG cells with similar effects observed. (**C**) Green fluorescent glucose analogue (NBDG, 200 μM) was perfused through the left lateral microchannel and the diffusion profile was studied in the absence or presence of cells. The graph shows the NBDG diffusion profile across the central microchamber after 90 min, demonstrating that NBDG was able to penetrate through the collagen hydrogel. The diffusion profile slope was calculated in the absence of cells or in the presence of HCT-116 or U-251 MG cells. Scale bar is 400 μm.

The existence of an oxygen gradient across the microchamber was assessed using confocal time-lapse microscopy and a hypoxia-sensing dye (Image-iT Hypoxia Reagent, Life Technologies) that increases its fluorescence as oxygen tension inside the cell decreases. For both U-251 MG and HCT-116 cells, hypoxia-induced fluorescence gradually appeared during the first hours of the experiment (panels in Fig. 5AB). A steep gradient of increasing hypoxia was observed along the first 300 microns through the “tumour slice” before reaching a plateau where oxygen concentrations had dropped to levels where the hypoxia sensing dye was fully activated. By creating an interstitial flow of fresh media through the hydrogel (as explained in Fig. 2), oxygen levels were restored to the initial values. Interestingly, just 4 hours later the hypoxia levels again reached the values observed after 24 hours in cell culture (graphs; Fig. 5A). These results demonstrate that hypoxia is generated within the microdevice and that this can be monitored and quantified in real-time. Furthermore, the levels of hypoxia can be manipulated by inducing an interstitial fluid flow through the microdevice.

The possible creation of a glucose concentration gradient across the microdevice was analysed using the fluorescent glucose analogue 2-(*N*-(7-Nitrobenz-2-oxa-1,3-diazol-4-yl)Amino)-2-Deoxyglucose(NBDG). Glucose-free media supplemented with 200 μM NBDG was perfused through one of the lateral microchannels and confocal time-lapse images were captured to study the diffusion profile. In the absence of cells, NBDG rapidly diffused through the hydrogel and after 90 min, the difference in fluorescence intensity between the NBDG-perfused microchannel and the opposite channel was only about 30% (Fig. 5C). When the experiment was repeated with 40 million cells/ml embedded in the collagen hydrogel, a different response was observed for the HCT-116 and U-251 MG cells. In the case of U-251 MG cells, NBDG appeared to penetrate through the hydrogel at a similar rate as in the absence of cells with no significant difference in the NBDG gradient generated. This suggests that glucose was able to penetrate across the ‘viable rim’ and into the ‘necrotic core’ of the U-251 MG cells. This is consistent with glucose being able to diffuse further than oxygen and support anaerobic metabolism which is a feature of the tumour microenvironment *in vivo*. It also indicates that U-251 MG glucose uptake rate was not sufficient to significantly delay the penetration of NBDG. In contrast, a very different NBDG gradient was generated in HCT116 cells where a higher NBDG-fluorescence intensity near the NBDG-perfused lateral microchannel was observed. In this case the gradient increased by nearly fifty percent compared to the control or the U-251 MG cell experiments (NBDG slope in the presence of HCT-116 = 0.035%/μm vs 0.022%/μm).

### Visualisation and characterization of ROS and apoptosis in the microdevice

**Figure 6:**
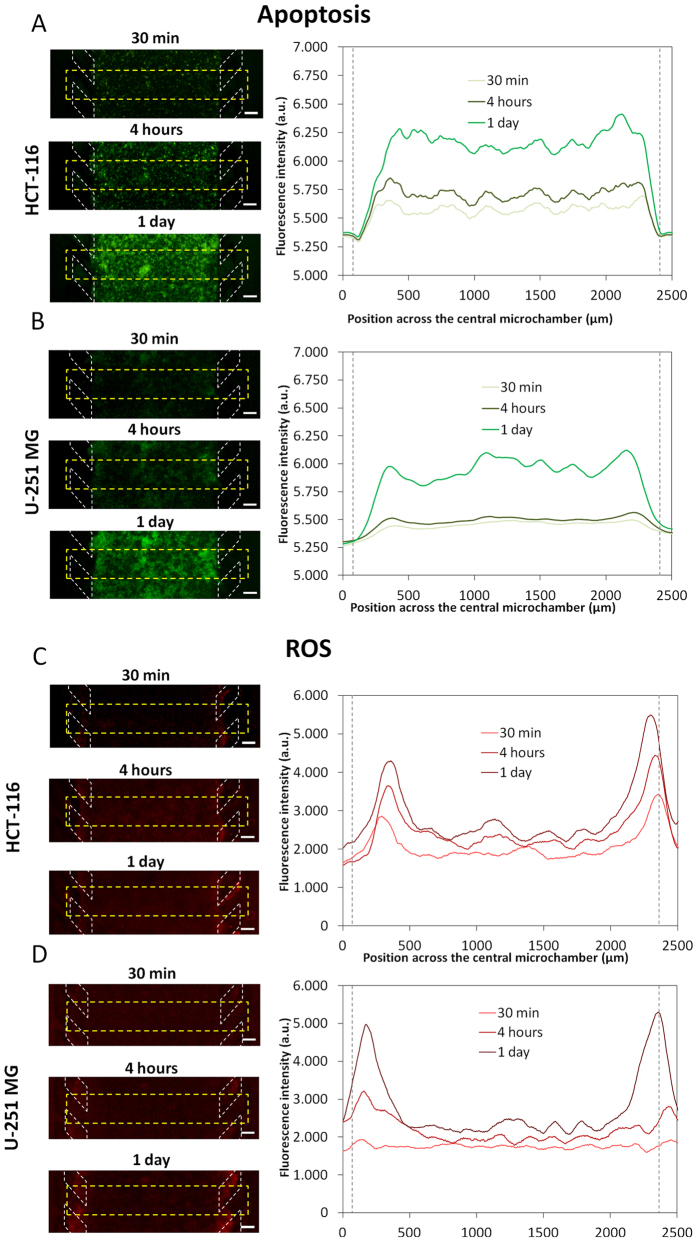
Apoptosis and ROS characterization. HCT-116 (**A**) and U-251 MG cells (**B**) were confined to the central microchamber of the device in hydrogel. An apoptosis sensing dye was perfused into the device via the lateral microchannels. For both cell lines, apoptotic levels were found to increase with time. The graphs show the apoptotic profiles across the delimited region. Position of the pillars is delimited by a grey dashed line. (**C,D**) For detection and monitoring of ROS production, a commercially available agent that is oxidised by ROS to a fluorescent product was perfused through the system. The graphs show quantification of ROS along the delimited region. Scale bar is 200 μm.

Following the demonstration that a microenvironment is generated within the microdevice characterized by hypoxia and formation of a central ‘necrotic core’, the capability of the microdevice to study and quantify the induction of apoptosis and reactive oxygen species (ROS) was investigated. Apoptosis was detectable with embedded U-251 MG as well as with HCT-116 cells, but apoptosis was not confined to the central ‘necrotic core’ region and was visible across the entire central chamber at similar levels (Fig. 6AB). This suggests that the PI-positive dead cells observed in the ‘necrotic core’ are likely to be necrotic rather than due to progression of early stage apoptotic cells into late stage apoptosis.

ROS levels across the device were also analysed. These were relatively low but highest at the “tumour slice” edges, overlapping with the more oxygenated areas shown in Fig. 5A (Fig. 6CD). The capability to quantify apoptosis and ROS in real-time in different regions of the ‘tumour slice’ is likely to be extremely useful both for basic research relating to the tumour microenvironment but also for drug response and mechanistic studies.

### Heterotypic cell interactions in the microdevice – migration of natural killer (NK) cells

The three chambers of the microdevice offer the opportunity to study heterotypic cell interactions and signalling as can occur in the actual tumour microenvironment. In order to demonstrate the capabilities of the microdevice to study cross-talk between tumour cells and the immune system, activated NK cells were perfused through one lateral microchannel, whereas GFP-expressing HC-T116 cells were confined in the central microchamber. Fluorescence time-lapse microscopy showed NK cells migrating from the lateral microchannel towards HCT-116 cells in the central microchamber with some NK cells penetrating into the “tumour-slice” (Supporting movie 4 and Supporting Fig. 5). This is a key advantage of the device compared to multicellular spheroid models, enabling real-time study of cell migration and dissection of whether an effect is due to direct heterotypic cell interactions or via heterotypic signalling.

### Drug penetration and evaluation of the impact of the microenvironment on drug responses

**Figure 7:**
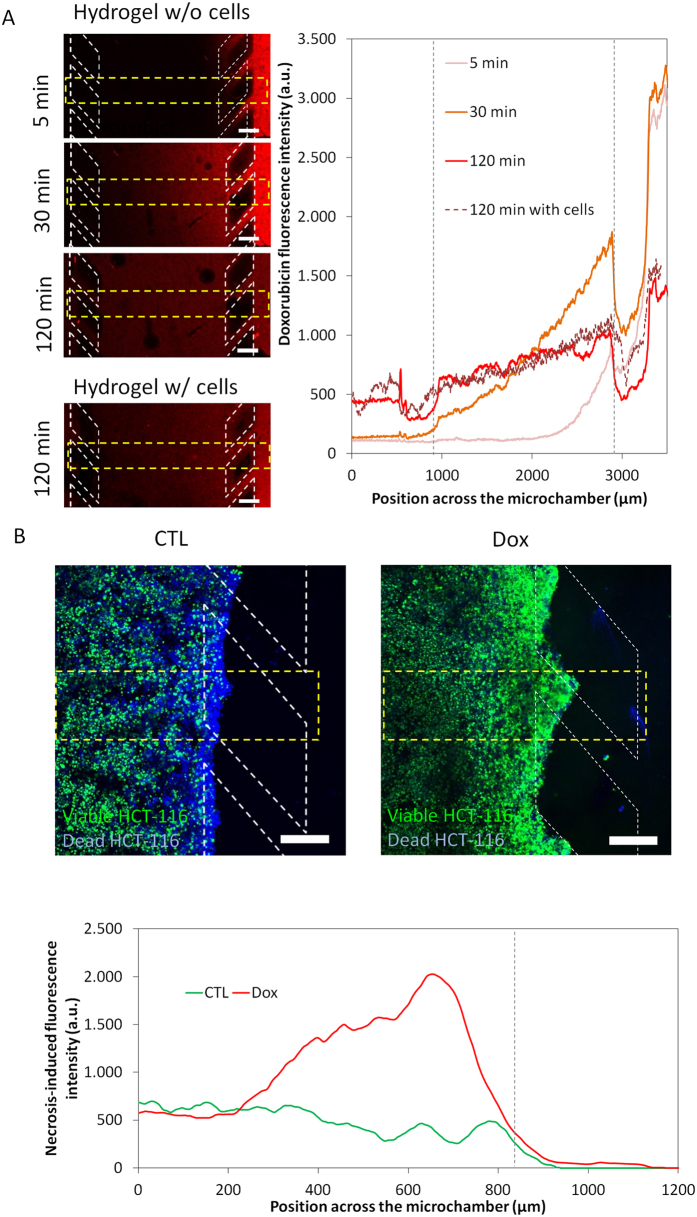
DOX effect on HCT-116 cells. The antiproliferative chemotherapy agent DOX was tested within the microdevice. (**A**) 100 μM DOX was perfused through one lateral microchannel to assess its penetration capacity through the hydrogel. The graph shows the DOX diffusion profile across the device with the time. Position of the pillars is delimited by a grey dashed line. (**B**) 30 μM DOX was perfused through both of the lateral microchannels to study the effects on cell viability. After 3 days in the presence of DOX, NucBlue® and NucGreen® dyes were perfused through the lateral microchannels. NucBlue® stains all cells blue whereas NucGreen® selectively stains dead cells green. The graph shows the strong effect of doxorubicin on HCT-116 cell mortality. The green fluorescence intensity was analysed along the delimited region. Scale bar is 200 μm.

There is an increasing need to study drug responses *in vitro* under more physiological conditions that reflect the complexity of the tumour microenvironment. Effective targeting of typically chemoresistant cells residing in hypoxic regions is a strategic priority. Another key consideration in evaluating drugs, for example in a multicellular spheroid, is whether a lack of response relates to the drug being ineffective or due to impaired drug penetration resulting in the inability of the drug to reach all cells in sufficient quantities to induce a response. Drug penetration through collagen hydrogel in the central chamber of the microdevice was evaluated using the cytotoxic drug doxorubicin (DOX), which is a DNA intercalating agent that naturally fluoresces red. DOX was perfused through one of the two lateral microchannels and confocal images were taken at different times, showing rapid penetration of the DOX (Fig. 7A). In the absence of cells, DOX concentration was almost linearly distributed across the microchamber after 2 hours and the concentration at the DOX-perfused channel and the opposite channel was only 30% different (Fig. 7A). When the experiment was repeated with 40 million HCT-116 cells/ml in the hydrogel and the addition of DOX 24 h later, DOX penetrated at a similar rate (Fig. 7A), demonstrating that DOX was reaching the hypoxic/necrotic region (as shown in Figs 3 and 5).

To study drug toxicity, DOX was perfused through both lateral microchannels and cell viability was assessed after 3 days. DOX induced significant HCT-116 cancer cell kill in cells located near to the lateral microchannels but induced much less cell kill further from the microchannels (Fig. 7B). For this experiment, DOX was found to efficiently penetrate into the hypoxic region suggesting that this difference in cytotoxic response may be due to a difference in the proliferative rate of the cells in these different regions (as has been shown in Fig. 4).

**Figure 8:**
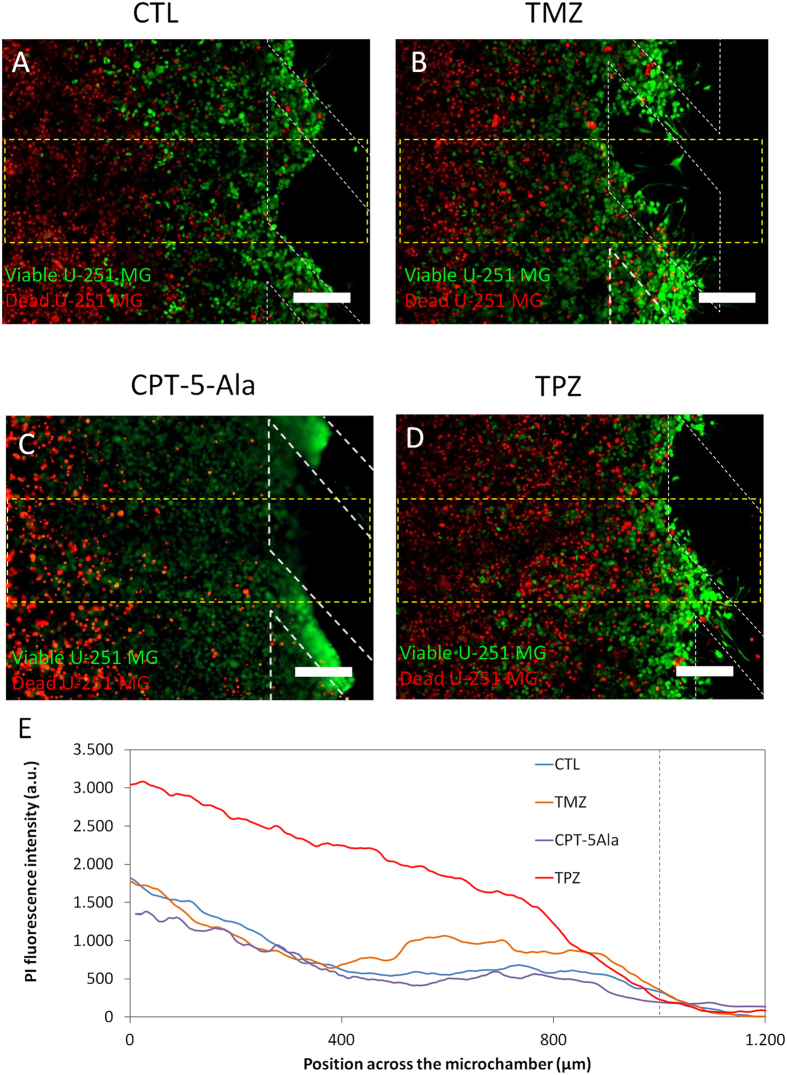
Drug effect on U-251 MG cells. U-251 MG cells were cultured within the central microchamber for 24 hours before drug addition in order to allow time for formation of a hypoxic region first. Growth medium (**A**), 100 μM TMZ (**B**), 1.6 μg/ml CPT-5Ala or 100 μM TPZ (**C**) were perfused through both lateral microchannels and after 3 days cell viability was assessed using by PI/CAM staining. (**E**) The graphs show the PI fluorescence intensity along the delimited region, demonstrating that TMZ had a mild effect on U-251 MG cell viability. TPZ exerted a strong effect in the hypoxic areas. Position of the pillars is delimited by a grey dashed line. Scale bar is 200 μm.

For Glioblastoma U-251-MG cells, the alkylating agent temozolomide (TMZ), which is in clinical use to treat Glioblastoma, was evaluated. For U-251 MG cells cultured in 2D for 7 days, TMZ significantly reduced viable cell number compared to controls (Supporting Fig. 6). As dead U-251 MG cells detached from the bottom of the Petri dish most of them were removed when the culture media was changed for viability staining, explaining the low apparent number of ‘red’ dead cells in the images. For cells seeded at the same cell density in 3D collagen hydrogels, cell proliferation over the same period was substantially reduced in 3D controls compared to 2D, indicating the impact of a 2D or 3D environment on cell growth. Consistent with a reduced proliferative rate and the mechanism of action of TMZ, which is dependent on DNA replication, TMZ had a much less dramatic effect on viable cell number for cells growing in hydrogel (Supporting Fig. 6). Indeed, TMZ had little effect compared to controls and just slightly higher levels of cell death were detected in the most oxygenated and more proliferative regions of the microdevice near the lateral microchannels (Fig. 8A,B,D).

A declining gradient of oxygen across the chamber provides the opportunity to evaluate potential hypoxia-targeting drugs or hypoxia-activated pro-drugs (HAPs). This is exemplified here using the HAP tirapazamine (TPZ). First, TPZ cytotoxicity was evaluated on cells embedded in 3D collagen hydrogels in Petri dishes that were cultured under normoxic (20% O_2_) or hypoxic (1% O_2_) conditions. Under normoxic conditions, 100 μM TPZ mildly reduced cell viability by less than 20% after 3 days, whereas 1 and 10 μM TPZ had no effect (Supporting Fig. 7). When cultured under homogenous hypoxic conditions, TPZ displayed a much more dramatic effect with cell viability reduced by ≈80% at 100 μM (Supporting Fig. 7). However, in the actual tumour microenvironment, cancer cells are exposed to a gradient of hypoxia with increasing distance from a blood vessel, which can be modelled in the microdevice (as has been shown in Fig. 5). U-251-MG cells were embedded within the microdevice and 24 h later, medium with 100 μM TPZ was perfused through the lateral microchannels and cell viability was measured 3 days later. TPZ cytotoxicity closely correlated with levels of hypoxia with cell mortality highest in the innermost most hypoxic regions and with increasing distance from the lateral microchannels (Fig. 8CD). In contrast, consistent with the activation of TPZ by hypoxia, those cells located near to the lateral microchannels, which are well oxygenated, remained viable (Fig. 8D).

## Discussion

In the last few decades our knowledge and understanding of cancer cell biology has improved considerably. However, the clinical approval of new drugs has not kept pace with this increased understanding of cancer biology. Many of the new drug candidates that show promise in initial testing fail when they are tested in more advanced models and the clinic^28^,^29^. One key strategy to reduce the high attrition rate and cost associated with the discovery of anti-cancer drugs is to develop new *in vitro* preclinical models that are better predictors of success in advanced preclinical and clinical testing^28^. Conventional two-dimensional cell cultures are unable to mimic many critical aspects of tumour biology (i.e. nutrient and waste gradients, hypoxia, gradients of cellular proliferative status), leading to drug failure in more advanced stages of the drug development process. New experimental *in vitro* models that can mimic key aspects of the tumour microenvironment and allow early assessment of effects of the tumour microenvironment on cellular drug response are urgently needed.

Technical complexity has hindered microfluidic-based models being widely adopted by the biomedical community. Here we present a very simple and easy-to-operate model that closely mimics important aspects of the tumour microenvironment and enables monitoring in real-time. Using this model, the two different cancer cell lines we analysed, HCT-116 and U-251 MG, generated quite different tumour microenvironments: (1) differing in the size of their ‘necrotic core’; (2) cellular rates of glucose uptake and oxygen consumption; and (3) their proliferative rates in different parts of the ‘tumour slice’ over time, particularly in the viable region nearest the ‘surrogate blood vessels’ (the lateral microchannels) (Figs 3, 4 and 5, Supporting Fig. 4). Due to their faster metabolism and proliferative rate, the HCT116 cells rapidly exhausted glucose and oxygen resulting in the formation of a necrotic region more quickly than the U-251 MG cells (Fig. 3). Formation of gradients of glucose and oxygen across the device were quantified in real-time (Fig. 5, Supporting Fig. 4). The ability to have a live visual ‘window’ through the ‘tumour slice’ as exemplified here by real-time monitoring of nutrients, viability and proliferation gradients highlights one of the key advantages of this model compared to the multicellular spheroids.

We also show that the microdevice can be used to measure other important factors such as levels of ROS and apoptosis in different parts of the ‘tumour slice’ in real-time. ROS are a product of cellular oxidative stress and a major cause of DNA damage and can, depending on circumstances, either promote cancer cell growth or be harmful to cancer cells^30^,^31^. In this context, the presented model could be used to study this phenomenon and the role of ROS in the mechanism of action of drugs that are being tested and their activity against cells in different parts of the microenvironment. In this study, in the absence of any drug treatment, ROS levels increased with culture time in the microdevice but were only detected near the “tumour-slice” edges, where oxygen was available.

Evasion of cell death by apoptosis is a major mechanism by which cancer cells survive and evolve and is a major target in the development of new drugs. We foresee a major potential application of the microdevice will be in the screening of new drug candidates and whether they are able to induce apoptosis as part of their mechanism of action and, importantly, in which cells within the modelled tumour microenvironment. Here, we demonstrate that apoptosis can be quantified in real-time through the microdevice, our results showing that basal apoptotic levels increased through the ‘tumour slice’ with culture time. Interestingly, there was little variation in different regions of the ‘tumour slice’ indicating that generation of the ‘necrotic core’ was not associated with apoptosis.

As a preliminary evaluation of the potential of the microdevice for *in vitro* drug testing that assesses the effects of different aspects of the tumour microenvironment upon drug response, three well-established drugs were tested. DOX is a naturally red fluorescing compound in clinical use against colon cancer and other cancers that intercalates DNA and is cytotoxic towards actively proliferating cells. Our results showed that DOX was most cytotoxic against cells located at the hydrogel edges near the lateral microchannels, where nutrients were available allowing rapid cell proliferation. As DOX fluoresces red, we were also able to analyse the ability of doxorubicin to penetrate through the ‘tumour slice’ at the cell density used. Here, we show that at the cell density used, DOX was able to efficiently penetrate across the device indicating that the preferential cytotoxicity observed was due to the mechanism of action of DOX as opposed to issues of DOX reaching all cells. Drug penetration barriers for doxorubicin have been described in spheroids and other 3D models^32^. However, in our model the inclusion of the ECM implied a reduction of the cell density compared to multicellular spheroids; explaining the enhanced drug penetration. Interestingly, this barrier effect could be mimicked in our model by increasing the cell density injected. Furthermore, the possibility of creating interstitial fluid flow could be used to explore new alternatives to increase the drug penetration into the tumours^33^. In contrast TPZ, which is activated to a toxic radical by low oxygen, was most cytotoxic towards those cells in the innermost hypoxic regions furthest from the lateral microchannels. This indicates the suitability of the microdevice to assess drugs and their activity towards cells in parts of the tumour microenvironment (e.g. well-oxygenated, nourished vs hypoxic). Although TPZ caused an increased in cell mortality in hypoxic regions, it was unable to destroy the tumour cells located in the “normoxic” areas within the “tumour-slice” model. Combinatorial therapies based on the use of different drugs could be good alternatives to kill cells in different parts of the tumour microenvironment and could also be easily assessed using the microdevice.

In conclusion, we have described the development of a microfluidic-based assay that mimics different of the key features of the solid tumour microenvironment. This is a complex microenvironment that has a profound effect on the outcome of chemotherapy and incorporating models of this nature into drug discovery procedures may help reduce the high attrition rate. This model is technically simple and in contrast to other three dimensional models such as multicellular spheroids, it provides a ‘window’ through which the effects of the microenvironment of tumour biology, heterotypic cell interactions, drug delivery and response to therapy can be observed and recorded in real-time. The model is inherently flexible and it offers the distinct advantage of being able to create microenvironmental conditions in cell lines that do not readily form three dimensional structures *in vitr*o. Additionally, culture medium could be retrieved from the microdevices in order to perform a more quantitative analysis of the chemotactic factors secreted by the cells. Finally, the microfluidic device described in this manuscript could reduce the number of animals required for *in vivo* testing as it could be positioned between traditional cell culture based evaluation and *in vivo* testing in preclinical drug development with only those compounds showing promising activity in the microfluidic model progressing onto *in vivo* testing.

## Methods

### Microdevice fabrication

Polystyrene-based microdevices were designed in order to allow user-friendly operation, avoiding the need for specialized clean-room processing equipment. The microdevice geometry included a 2000 μm wide central microchamber flanked by two 700 μm wide lateral microchambers with the depths of the chambers being 250 μm. The central microchamber was delimited by parallelogram-shaped pillars that allowed liquid and cell confinement within hydrogels as described elsewhere^23^. Lateral microchannels remained hydrogel-free and were perfused with media or drug-containing media. Dedicated inlets and outlets were also integrated to allow liquid or hydrogel injection by manual pipetting. The configuration of the microdevice is illustrated in Fig. 1A. Prior to their use in cell culture, microdevices were sterilized by submerging in 70% ethanol. After air-drying and 2 hours UV exposure, microdevices were ready for use.

### Reagents

Temozolomide (TMZ) (Sigma, T2577), Doxorubicin (DOX) (Selleckchem, S1208), Tirapazime (TPZ) (Sigma, SML0552) and Image-iT Hypoxia Reagent (Life Technologies, H10498) were dissolved in DMSO at 100, 100, 50 and 1 mM respectively. Image-iT Hypoxia Reagent was used in cell culture at 10 μM, whereas DOX, TPZ and TMZ were used at varying concentrations (the final DMSO concentration used was <0.1%). The CellEvent™ Caspase-3/7 Green Detection Reagent (Thermo, R37111) and the CellROX® Orange Reagent (Thermo, C10443) were used to detect apoptosis and ROS production respectively following supplier instructions.

### Prodrug synthesis

A commercial camptothecin (CPT) novel derivative was prepared by esterification of 5-aminolevulinic acid (5-Ala). For this purpose, N-tert-butyloxycarbonyl-5-aminolevulinic acid (N-boc-5-Ala) was synthesized by reaction of di-tert-butyl dicarbonate with 5-Ala. Then, this product was esterified on 20(S)-hydroxyl 20 position of CPT, followed by deprotection of the amine group with trifluoroacetic acid, obtaining camptothecin-20(S)-5-aminolevulinate (CPT-5-Ala) of 90% purity according to HPLC-MS/MS, ^1^H-NMR and ^13^C-NMR analysis (Supporting Fig. 1). The Camptothecin prodrug (CPT-5-ALA) was dissolved in DMSO and perfused through the microdevices at 1.6 μg/ml.

### Characterisation of flow

In order to study fluid flow through the microdevice, 0.2 μm-diameter green-fluorescent microsphere beads (Life Technologies, F8811) were diluted 1/100 in growth medium and perfused through the microdevice by manual pipetting.

### Cell culture

Human cancer cell lines HCT-116 (colon carcinoma), U-251 MG (Glioblastoma) and Jurkat (leukaemia) were purchased from the American Type Culture Collection and routinely cultured in high glucose Dulbecco’s modified Eagle’s medium (DMEM) (Lonza, BE12-614F) supplemented with 10% v/v foetal bovine serum (FBS) (Sigma, F7524, non-USA origin), 2 mM L-glutamine (Lonza, 17-605C) and penicillin/streptomycin (Lonza, DE 17-602E) within a TEB-1000 humidified 5% CO_2_ incubator (EBERS Medical Technology) at 37 °C. The R69 lymphoblastoid B cell line was kindly provided by Dr López de Castro, from the “Severo Ochoa” Molecular Biology Centre (CBMSO) and were cultured in RPMI1640 (Sigma, R0883) supplemented with 10% FBS and L-glutamine.

### Isolation and activation of human NK cells

Human NK cells were derived from the blood of healthy donors provided by the Blood and Tissue Bank of Aragon. After Ficoll gradient centrifugation of whole blood, freshly isolated peripheral blood mononuclear cells (PBMCs) were activated *in vitro* for 21 days. PBMC cultures were maintained in RPMI-1640 medium (Lonza) supplemented with 10% FBS (HyCloneTM); 100 U/ml IL-2 (Miltenyi) and 5 ng/ml IL-15 (Miltenyi), mitomycin C-inactivated R69 cells at a 10:1 ratio (PBMC:stimulator cells); penicillin/streptomycin and glutamine. Culture medium was renewed on the 7^th^ day followed by successive reactivation every 3 days to establish a cellular density of 1 × 10^6^ PBMCs/ml. Magnetic immune-separation using anti-CD56 antibodies (MACS, Miltenyi) was used to enrich for activated NK cells. The quality of NK cell activation was assessed by testing their cytotoxic potential against Jurkat cells as described previously^34^. Activated NK cells were fluorescently labelled with 3 μM eFluor670 (eBioscience; 65-0840) for 15 min to enable their discrimination from the tumour cells during the experiments. All protocols related with human samples were approved by the Ethical Committee of Clinical Research of Aragon (number: C.I.PI.11/006) and informed consent was obtained from all patients. Experiments were performed in accordance with the Spanish research regulations.

### Fluorescent cell labelling

Dio and Dil Vybrant® lipophilic cell membrane dyes (Life technologies, V-22886 and V-22885) were used to fluorescently label cells green or red respectively as per the manufacturer’s instructions. Briefly, after cell trypsinization, 5 μl of Vybrant® solution was added to 1 ml of cell suspension (10^6^ cells/ml) and incubated for 5 min. Cell suspensions were then centrifuged and washed twice with growth medium.

### Stable cell transfection with EGFP

U-251 MG and HCT-116 were stably transfected with EGFP using lentiviral vectors, kindly provided by Dr Prats, University Paul Sabatier, Toulouse, France^35^. Briefly, 5 × 10^4^ cells/well were seeded in 24-well plates and incubated for 24 hours at 37 °C in a humidified atmosphere containing 5% CO_2_. Growth medium was then removed and cells were washed twice with PBS (Lonza, BE17-516F). Protamine supplemented-OptiMEM (5 μg/ml, Sigma-Aldritch, P4020) was mixed with the lentivirus suspension in a 1:1 ratio and this mixture was added to the cells. After 24 hours, transfection medium was replaced with growth medium and the cells were routinely cultured for two weeks in order to remove the viral particles. Transfection efficiency was checked by fluorescence microscopy and flow cytometry (BD, FACSAria) with more than 90% of the cells found to be EGFP-positive.

### Spheroid generation

U-251 MG and HCT-116 multicellular spheroids were generated by the hanging drop method, supplementing the culture medium with methylcellulose^22^. Briefly, 6g of high viscosity methylcellulose (Sigma, M0512) was dissolved in 500 ml of high glucose DMEM to make a stock solution. Cells were trypsinized, counted and cell suspensions were generated at 5 × 10^4^, 5 × 10^5^, 5 × 10^6^ cells/ml. These different cell suspensions were then mixed with the methylcellulose stock solution in a 4:1 ratio. Finally, 25 μl droplets (1 × 10^3^, 1 × 10^4^ or 1 × 10^5^ cells/droplet) were placed onto the lid of Petri dishes with sterile water added to the bottom in order to reduce droplet evaporation and incubated at 37 °C and 5% CO_2_ for 24 h. Using this methodology a single well-defined spheroid of a particular size was generated per droplet.

### 3D cell culture within the microdevices

For preparation of three-dimensional cell cultures in collagen hydrogel, all reagents and microdevices were placed on ice. Cells were trypsinized and re-suspended in a calculated volume of growth medium to reach the desired cell concentration (4 × 10^6^, 10 × 10^6^ or 40 × 10^6^ cells/ml) in the final hydrogel solution. Using a chilled tip, a mixture of 24.9 μl collagen type I (4.01 mg/ml, Corning 354236); 0.62 μl NaOH 1N (Sigma 655104), 10 μl DMEM 5X (Sigma D5523), 50 μl cell solution and 14.5 μl sterile water was prepared. Hydrogel mixture was injected into the microfluidic device using a micropipette and a 10 μl droplet was placed on top of the inlet to prevent hydrogel evaporation. Afterwards, the microfluidic device was placed into an incubator (37 °C and 5% CO_2_) for 15 minutes to allow collagen polymerization. 5 ml of culture medium was added to cover all the microdevices attached to the Petri dish. Culture medium was perfused through the lateral microchannels to allow oxygen and nutrient diffusion. In all experiments, culture medium in the lateral microchannels was refreshed once a day by pipetting 10 μl through each lateral microchannel (unless otherwise stated). U-251 MG and HCT-116 cells were viable within the microdevice for all the different cell densities assessed after 6 days in cell culture. When a different culture medium (e.g. medium supplemented with drugs) was desired, culture medium was removed from the Petri dish by aspiration, 5 ml of the new medium was added and 10 μl were perfused through the lateral microchannels by manual pipetting.

### Cell proliferation measurement

Cell proliferation was observed in live cells using the Premo™ FUCCI Cell Cycle Sensor (Thermo, P36237). Briefly, cells were transduced using a 50 virus particle/cell ratio for 48 hours. Transduced cells were trypsinized and cultured in 3D within the microdevice as described above. This cell cycle sensor was transduced into the cells using two different reporters coupled to TagRFP or emGFP that are expressed alternatively during the G1 phase or the S/G2/M phases respectively.

### Cell viability analysis

Stock solutions of 5 mg/ml Calcein (CAM) (Life Technologies, C1430) and 2 mg/ml propidium iodide (PI) (Sigma P4170) were dissolved in DMSO and distilled water respectively. To test cell viability within microfluidic devices and in Petri dishes, stock solutions of CAM and PI were diluted to 5 and 4 μg/ml, respectively, in phosphate-buffered saline (PBS) (Lonza BE17-516F). CAM/PI solution was perfused through the lateral microchannels. Cells were visualized by confocal microscopy (Nikon Ti-E coupled to a C1 modular confocal microscope), with viable cells (CAM-positive) staining green and dead cells (PI-positive) staining red. Cell viability profiles were evaluated by analysing the fluorescence intensity of the viable/dead cells across the central microchamber. All confocal images were taken at different focal planes with subsequent image analysis performed using FIJI software. DOX and PI naturally fluoresce red; therefore an alternative cell viability staining solution was used during DOX experiments: 50 μl of NucGreen® Dead 488 ReadyProbes® Reagent (Life technologies, R37109) and 50 μl of NucBlue® Live ReadyProbes® Reagent (Life technologies, R37605) were added to 900 μl of growth medium. This solution was perfused through the lateral channels, labelling all the cells in blue and dead cells in green.

### Drug penetration assays

In order to study the penetration of soluble drugs through the microdevice, a 1.2 mg/ml collagen hydrogel was confined in the central microchamber. After collagen polymerization, 100 μM DOX, dissolved in culture medium, was injected through one lateral microchannel. Given the red fluorescence of DOX, confocal images were taken at different times to monitor the DOX penetration. Next, to determine whether the cells hinder the drug penetration, the experiment was repeated confining a collagen hydrogel with 40 million HCT-116 cells/ml embedded.

### Drug toxicity assays

HCT-116 cells were confined at 40 million cells/ml in a 1.2 mg/ml collagen hydrogel. After six hours in culture, 30 μM DOX, dissolved in culture medium, was injected through both lateral microchannels. Cell viability was evaluated after 3 days in culture.

Prior to any study within the microdevices, the effect of TMZ and TPZ was evaluated on conventional Petri dishes. 50 μM, 100 μM and 200 μM TMZ was assessed on U-251 MG cells cultured in 2D, as well as in 3D collagen hydrogels in Petri dishes. U-251 MG cells were incubated in the presence of TMZ and the total number of viable cells was determined after 1, 3 and 7 days. The effect of 1, 10 and 100 μM TPZ on cell viability was evaluated in 3D collagen hydrogels in Petri dishes cultured under 20% or 1% O_2_. Cell viability was evaluated after 3 days.

U-251 MG cells were cultured at 40 million cells/ml embedded in a 1.2 mg/ml collagen hydrogel confined in the central microchamber. After six hours in culture, 100 μM TMZ, 1.6 µg/ml CPT-5-Ala or 100 μM TPZ (all of them dissolved in culture medium) was injected through both lateral microchannels and cell viability was evaluated after 3 days.

### Image analysis

Laser confocal and fluorescence images were acquired using a Nikon Eclipse Ti-E C1 confocal microscope. The microscope has a temperature and CO_2_ controlled environmental chamber, enabling live cell and time-lapse imaging. Temperature and CO_2_ were set at 37 °C and 5% respectively. Images were analyzed using Fiji® software ( http://fiji.sc/Fiji). Fluorescence intensity across the central microchamber of the microdevice was quantified in the different experiments by selecting a rectangular region across the central microchamber. The fluorescence intensity across that section was then determined using the Fiji® software in accordance with the software instructions. In order to quantify cell proliferation, proliferating cell were manually counted in the different vertical regions of the central microchamber. In TMZ and TPZ experiments in Petri dishes, cell viability was determined by manual counting of viable and dead cells and calculating the percentage of viable cells relative to the total number of cells.

### Statistical analysis

All the experiments were repeated at least three times as independent biological repeats. Drug toxicity study results are presented as the mean ± standard error. The normal distribution was tested by the Kolmogorov-Smirnov test. Statistical significance was set at p < 0.05. For nonparametric comparisons, a Kruskal-Wallis test was performed followed by the Mann-Whitney U-test.

## Additional Information

**Publisher's note**: Springer Nature remains neutral with regard to jurisdictional claims in published maps and institutional affiliations.

**How to cite this article**: Ayuso, J. M. *et al.* Development and characterization of a microfluidic model of the tumour microenvironment. *Sci. Rep.* **6**, 36086; doi: 10.1038/srep36086 (2016).

## Supplementary Material

Supplementary Movie 1

Supplementary Movie 2

Supplementary Movie 3

Supplementary Movie 4

Supplementary Information
