# Supplementary material for: Development and characterization of a microfluidic model of the tumour microenvironment
Source: Sci Rep. 2016 Oct 31;6:36086. doi: 10.1038/srep36086 (PMC5086897; doi:10.1038/srep36086)
Supplement: Supplementary Information [file srep36086-s5.pdf]

Development and characterization of a microfluidic model of the tumour microenvironment.

Jose M Ayuso[1,2,3],María Virumbrales-Muñoz[1,2,3], Alodia Lacueva[1,2,3], Pilar M Lanuza[4,5], Elisa Checa-Chavarria[6,7], Pablo Botella[8], Eduardo Fernández[6,7], Manuel Doblare[1,2,3], Simon J Allison[9], Roger M Phillips[10], Julián Pardo[4,5,11,12], Luis J Fernandez[1,2,3*], Ignacio Ochoa[1,2,3*]

1 Group of Structural Mechanics and Materials Modeling (GEMM). Centro Investigacion Biomedica en Red. Bioingenieria, biomateriales y nanomedicina (CIBER-BBN), Spain.
2 Aragón Institute of Engineering Research (I3A), University of Zaragoza, Spain.
3 Aragon Institute of Biomedical Research, Instituto de Salud Carlos III, Spain.
4 Aragón Health Research Institute (IIS Aragón), Biomedical Research Centre of Aragón (CIBA), Zaragoza, Spain
5 Dpt. Biochemistry and Molecular and Cell Biology, University of Zaragoza, Zaragoza, Spain.
6 Centro Investigacion Biomedica en Red. Bioingenieria, biomateriales y nanomedicina (CIBER-BBN), Spain.
7 Bioengineering Institute. University Miguel Hernández, Spain.
8 Instituto de Tecnología Química (Universitat Politècnica de Valencia-Consejo Superior de Investigaciones Científicas), Spain
9 Department of Biology, University of Huddersfield, Queensgate, Huddersfield HD1 3DH, United Kingdom.
10 Department of Pharmacy, University of Huddersfield, Queensgate, Huddersfield HD1 3DH, United Kingdom.
11 Dpt. Microbiology, Preventive Medicine and Public Health, University of Zaragoza, Zaragoza, Spain.
12 Aragón I+D Foundation (ARAID), Government of Aragon, Zaragoza, Spain
*These authors equally coordinated this project.
Corresponding authors: Luis Fernandez-luisf@unizar.es. Ignacio Ochoa-iochgar@unizar.es

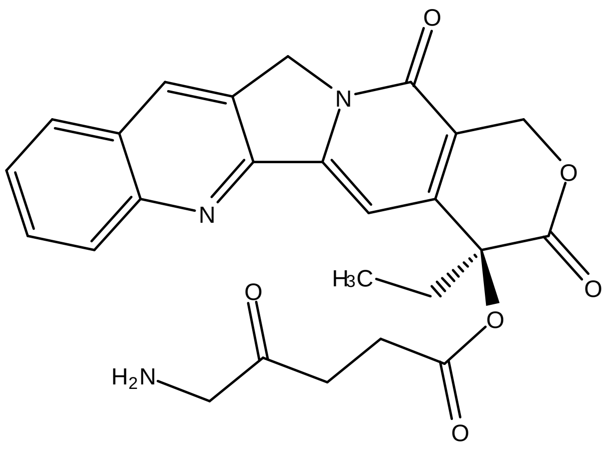

Supporting Figure 1. Molecular formula of synthesized camptothecin-20(S)-5-aminolevulinate.

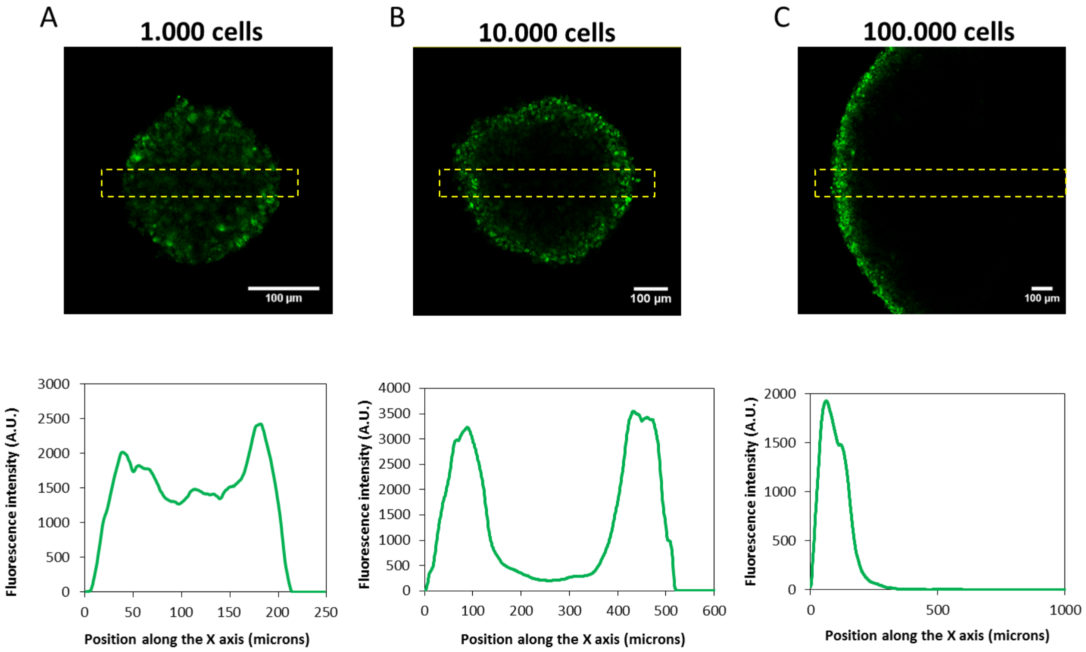

Supporting Figure 2. Impact of spheroid diameter on detecting fluorescently labelled cells. Dio-labelled U-251 MG cell spheroids. As the spheroid size increases, the visible region compared to the spheroid diameter is reduced (A- 1000 U-251 cells/spheroid, B- 10000 U-251 cells/spheroid, C- 100000 U-251 cells/spheroid). Graphs show the fluorescence intensity across the delimited region.

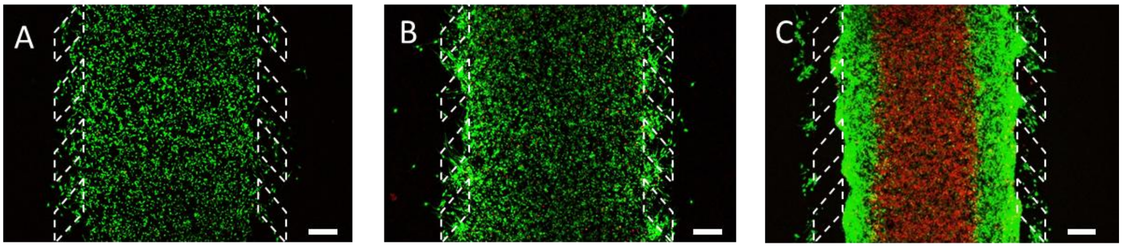

Supporting Figure 3. Generation of a necrotic core depends on cell density. U-251 MG cells were embedded within the collagen hydrogel at different cell densities (4, 10 and 40 million cells/ml in A, B and C respectively) and were cultured for 3 days. Cell viability was evaluated by calcein (CAM) and propidium ioidide (PI) staining. A necrotic region appeared by 3 days for the highest cell density of U-251 MG cells seeded but not for lower cell densities. Scale bar is 400μm.

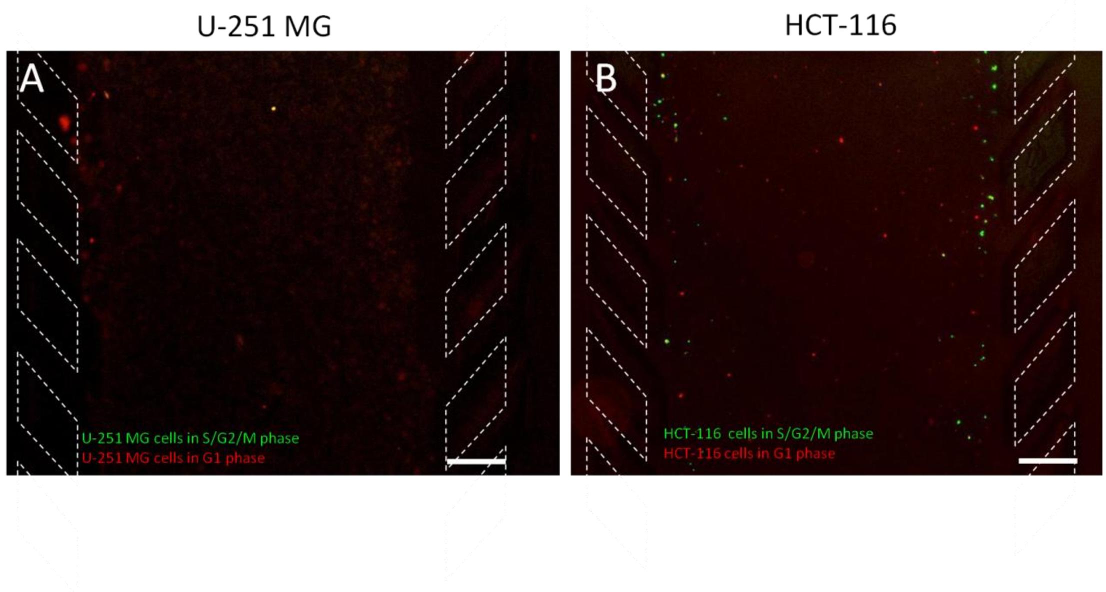

Supporting Figure 4. Cell proliferation. 40 million of U-251 MG (A) or HCT-116 (B) cells/ml were confined to the central microchamber; 5% of these cells were transduced with the cell cycle sensor Premo FUCCI®. After 24 hours, fluorescence images were taken, showing G1 phase and quiescent cells in red, and S, G2 and M phase cells in green. Scale bar is 400 microns.

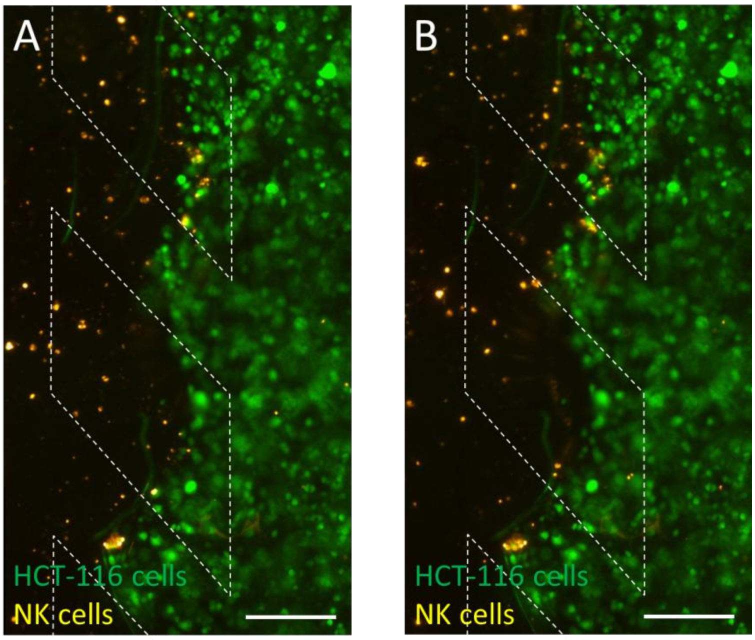

Supporting Figure 5. NK-HCT-116 co-culture. GFP-HCT-116 cells were embedded in a 1.2 mg/ml collagen hydrogel within the central microchamber. After collagen hydrogel polymerization, eFluor670-labelled NK cells were perfused through one of the lateral microchannels and images after 30 min (A) and 100 min (B) are shown. Some NKs cells have migrated towards the HCT116 cancer cells and have penetrated into the hydrogel by both time points. Scale bar is 200µm.

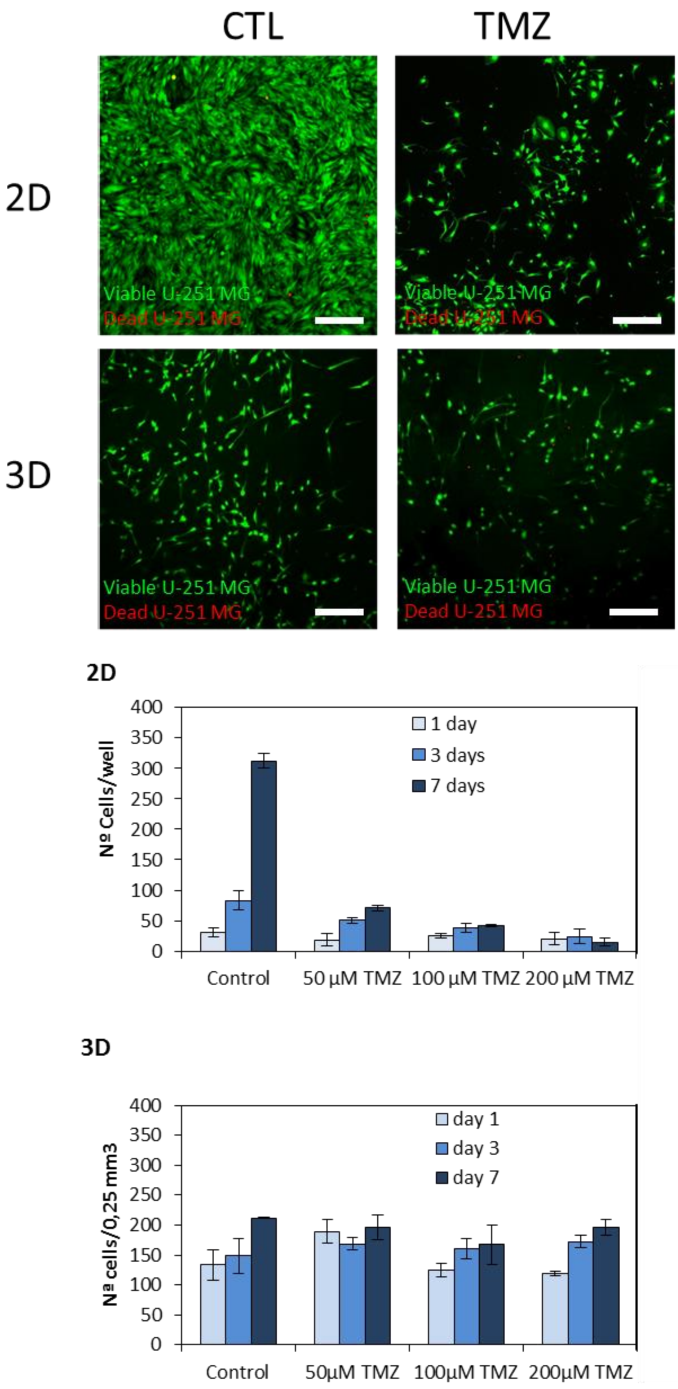

Supporting Figure 6. TMZ effect. U-251 MG cells were cultured in 2D in Petri dishes or embedded within 3D collagen hydrogels. After 24 hours, TMZ at different concentrations was added to the cultures. Cell viability was evaluated after 1, 3 and 7 days in cell culture using CAM and PI staining. The images show viable cells in green and dead cells in red after 7 days in culture. Incubation in the presence of TMZ for 7 days caused a dramatic reduction in the number of cells compared to controls for cells cultured in 2D. This reduction in cell number compared to control cells was much less for the cells that were cultured in 3D. Graphs show the number of viable cells at different time points in the different culture conditions. Scale bar is 100 µm.

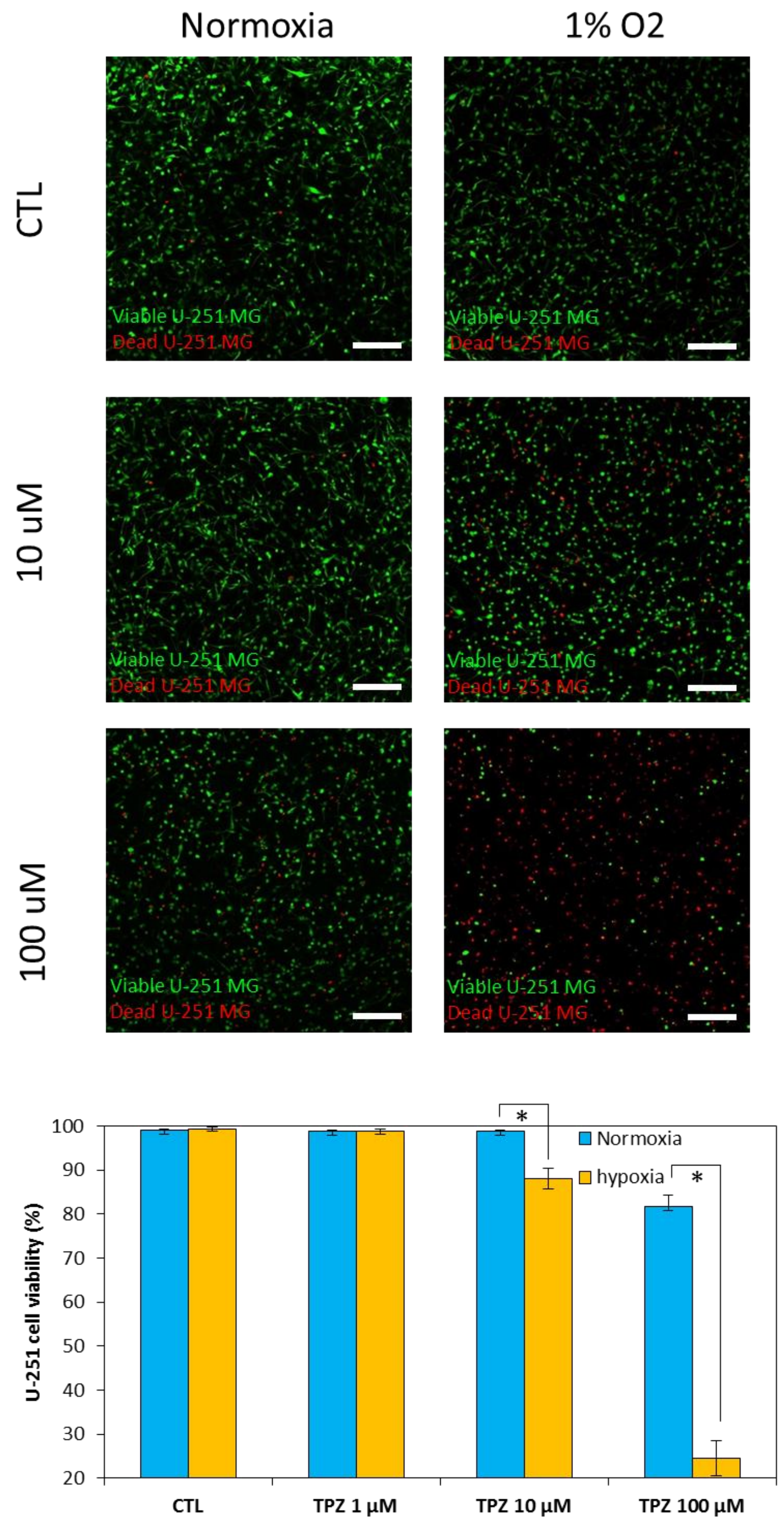

Supporting Fig 7. TPZ effect. U-251 MG cells were embedded within 3D collagen hydrogels. After 24 hours, TPZ at different concentrations was added and cells were cultured in normoxic (20% O_2) or hypoxic (1% O_2) conditions. After three days in cell culture, TPZ effect on cell viability was evaluated using PI/CAM staining. TPZ exerted a

stronger cytotoxic effect under hypoxic conditions; especially at 100 µM. Viable cells stain in green, whereas dead cells are stained red. Scale bar is 100 µm.

Supporting movie 1. Parallel flow. Green-fluorescent beads flowing through the left microchannel. Beads flow parallel to the hydrogel and no penetration is observed.

Supporting movie 2. Interstitial flow. Green-fluorescent bead flow. Beads penetrate into the hydrogel.

Supporting movie 3. HCT-116 necrotic core generation. The time-lapse movie shows the change in HCT116 cell viability following seeding in hydrogel in the central microchamber and the gradual formation of a necrotic core of HCT-116 cells during the first 24 hours l in culture. Cell seeding density was 40 million cells/ml and cell viability was detected using CAM and PI staining, which labels viable cells in green and dead cells in red respectively.

Supporting movie 4. NK-HCT-116 co-culture. GFP-HCT-116 cells were embedded in the central microchamber in collagen hydrogel. EFluor670-labelled NK cells were perfused through a lateral microchannel. The time-lapse movie shows NKs cells penetrating into the “tumour-slice”.
